# Supplementary material for: The Mycobacterium ulcerans toxin mycolactone causes destructive Sec61-dependent loss of the endothelial glycocalyx and vessel basement membrane to drive skin necrosis
Source: eLife. 2025 Feb 6;12:RP86931. doi: 10.7554/eLife.86931 (PMC11801798; doi:10.7554/eLife.86931)
Supplement: Figure 4—source data 2. — Immunoblots for Figure 4G were performed as described in the legend of Figure 4. This file shows the full-size blots probed with anti-dHS and anti-GAPDH antibodies for each of three biological repeats (‘2 reps’ and ‘3rd rep’), alongside molecular weight markers and annotated for treatments. [file elife-86931-fig4-data2.zip › Figure 4-source data 2/Figure 4G Annotated blots 3 reps.pdf]

dHS

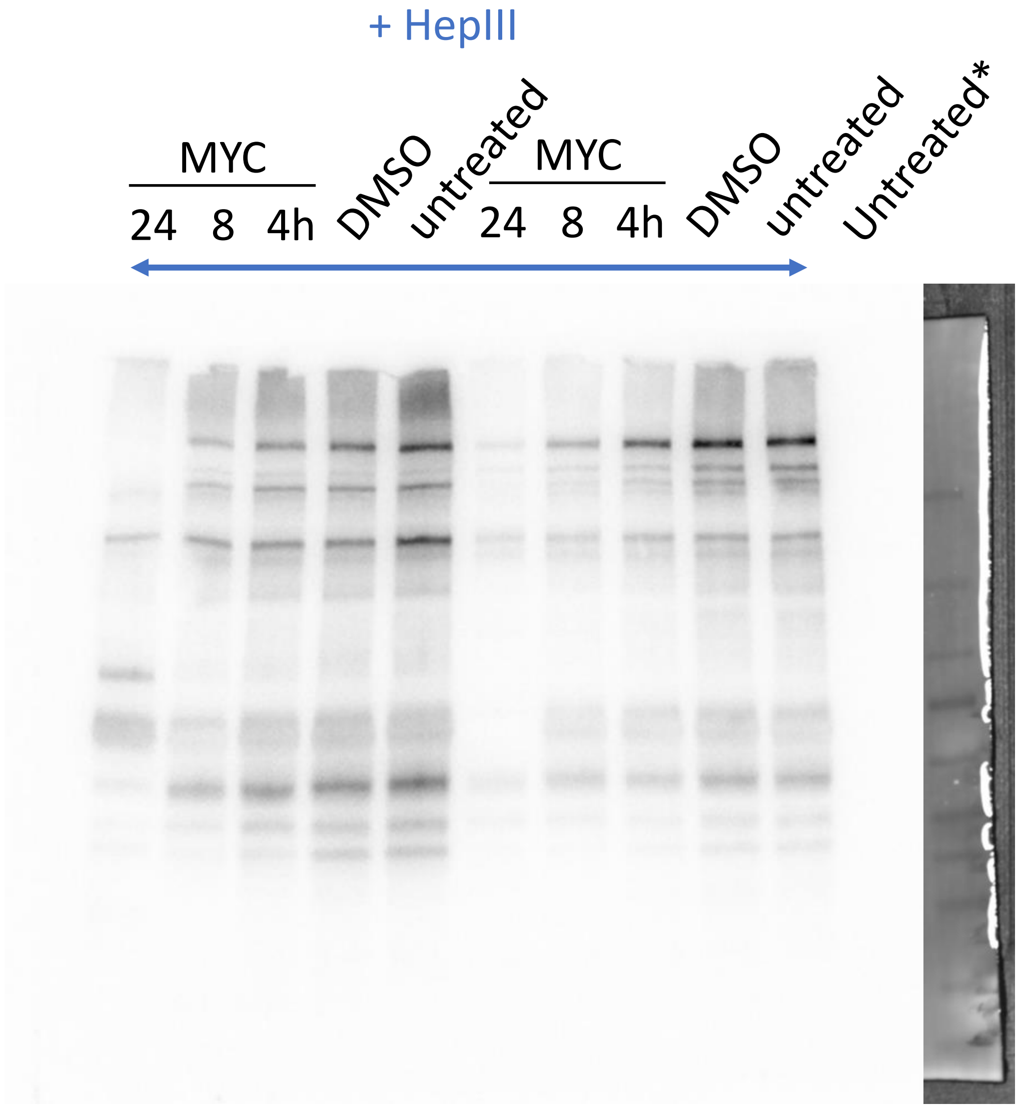

2 Reps

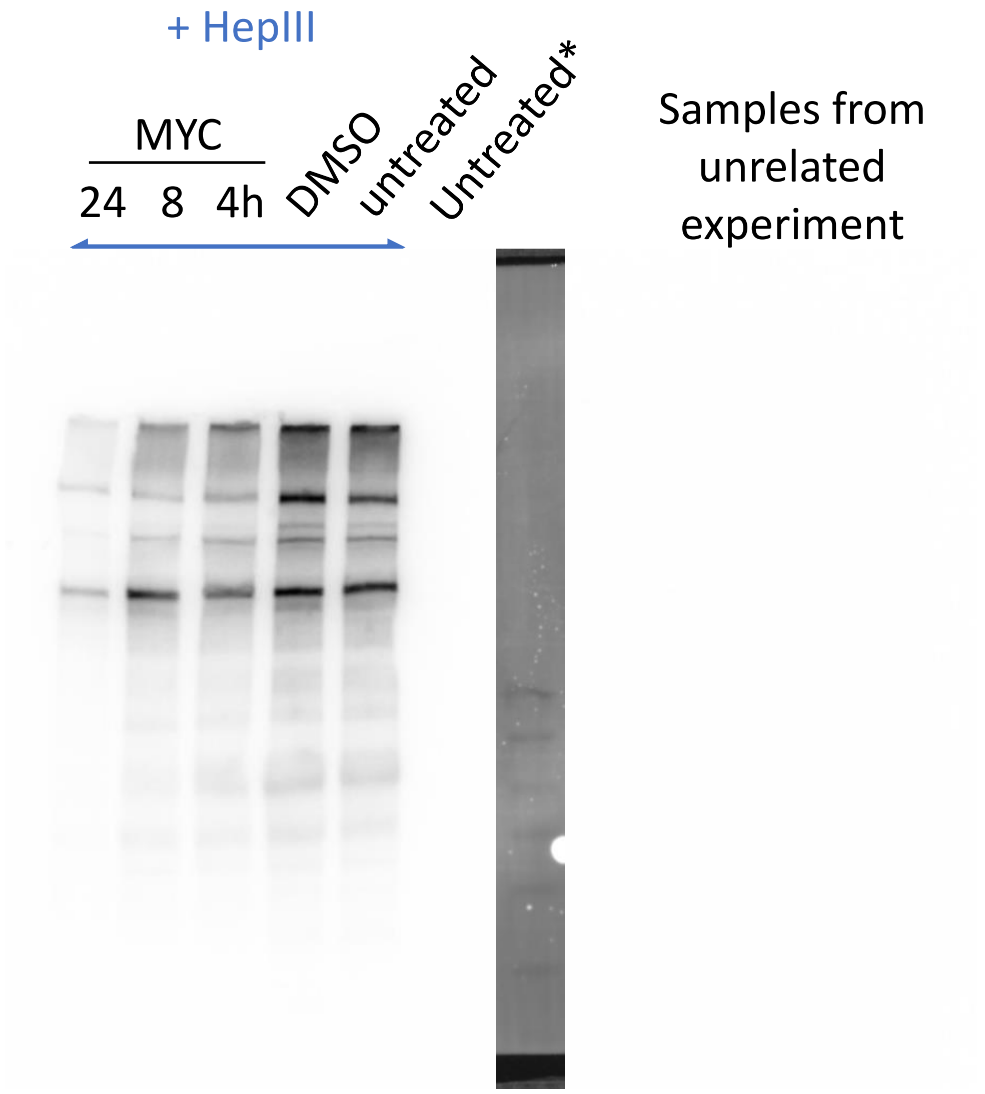

3rd Rep

* = control sample untreated with Heparinase III (HepIII): antibody control

GAPDH

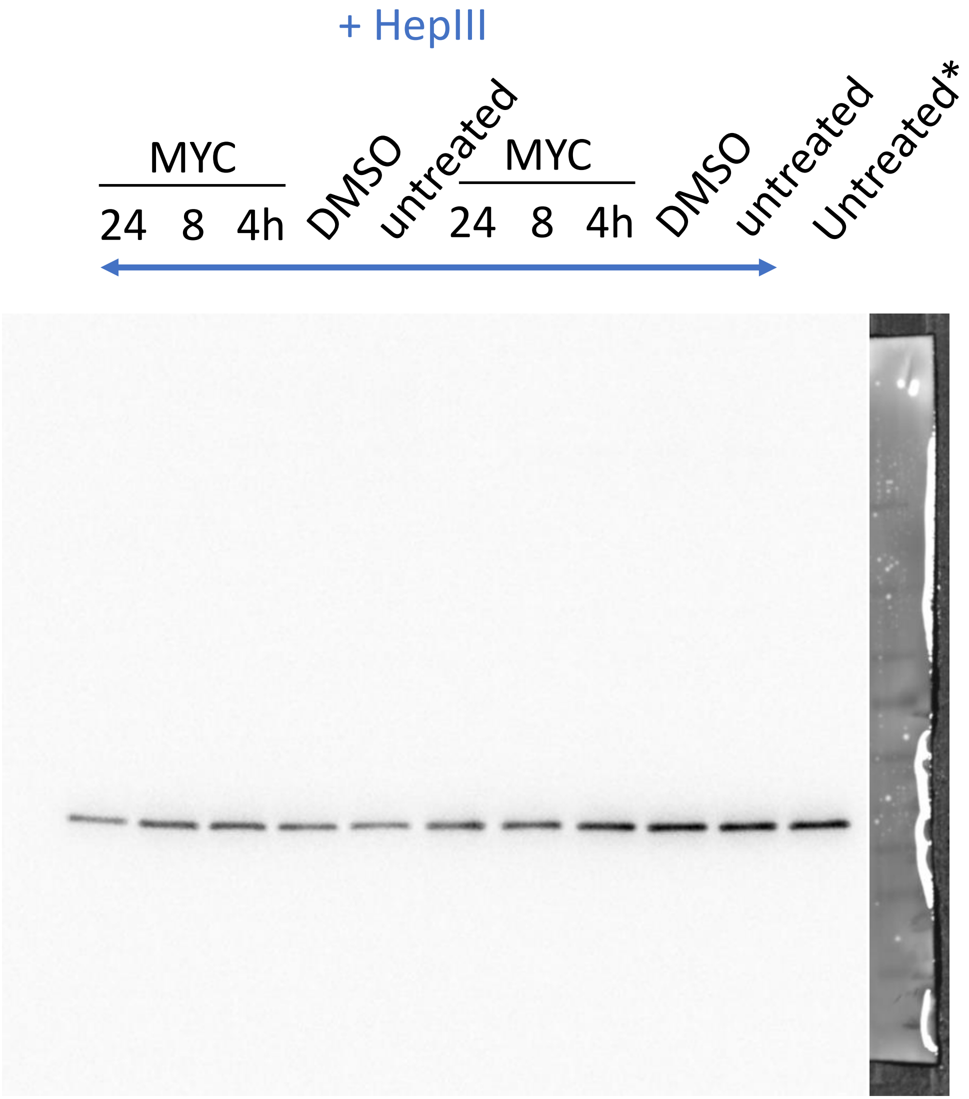

2 Reps

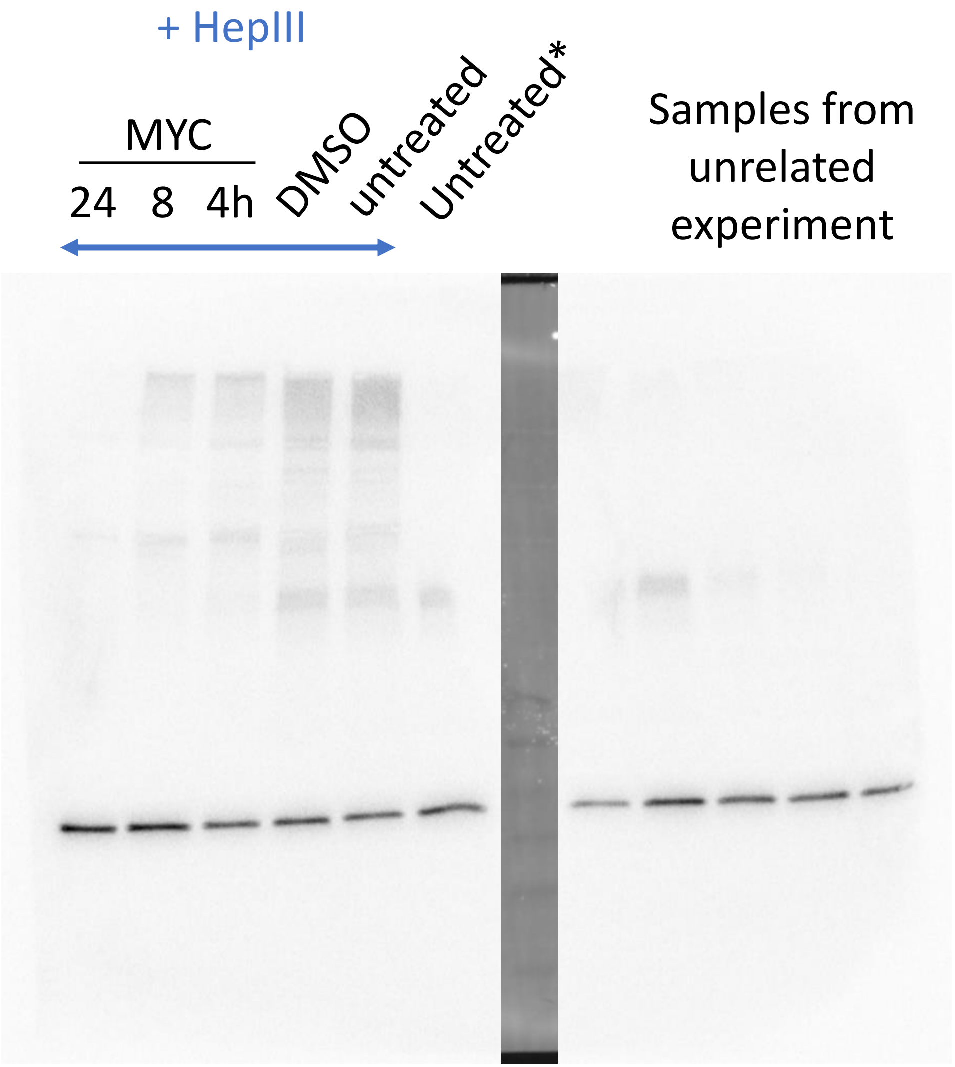

3rd Rep

* = control sample untreated with Heparinase III (HepIII): antibody control
