# Supplementary figures and images for: The Mycobacterium ulcerans toxin mycolactone causes destructive Sec61-dependent loss of the endothelial glycocalyx and vessel basement membrane to drive skin necrosis
Source: eLife. 2025 Feb 6;12:RP86931. doi: 10.7554/eLife.86931 (PMC11801798; doi:10.7554/eLife.86931)

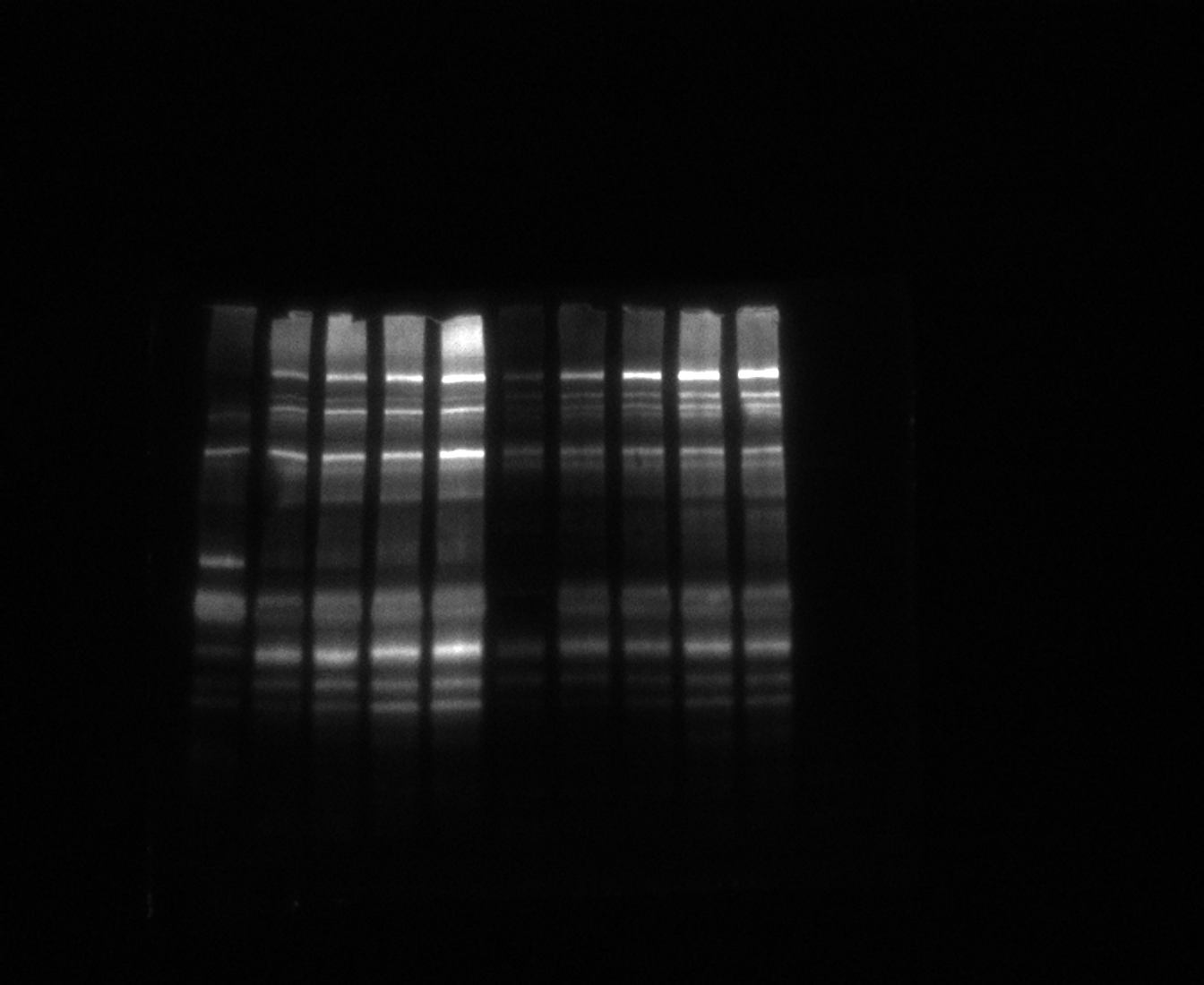

Supplement: Figure 4—source data 3. — Immunoblots for Figure 4G were performed as described in the legend of Figure 4. This folder contains the raw images collected on the Fusion FX Imager (Vilber-Lourmat) for blots probed with anti-dHS and anti-GAPDH antibodies for each of three biological repeats (‘2 reps’ and ‘3rd rep’). [file elife-86931-fig4-data3.zip › Figure 4-source data 3/HDMEC dHS (2 reps).Tif]

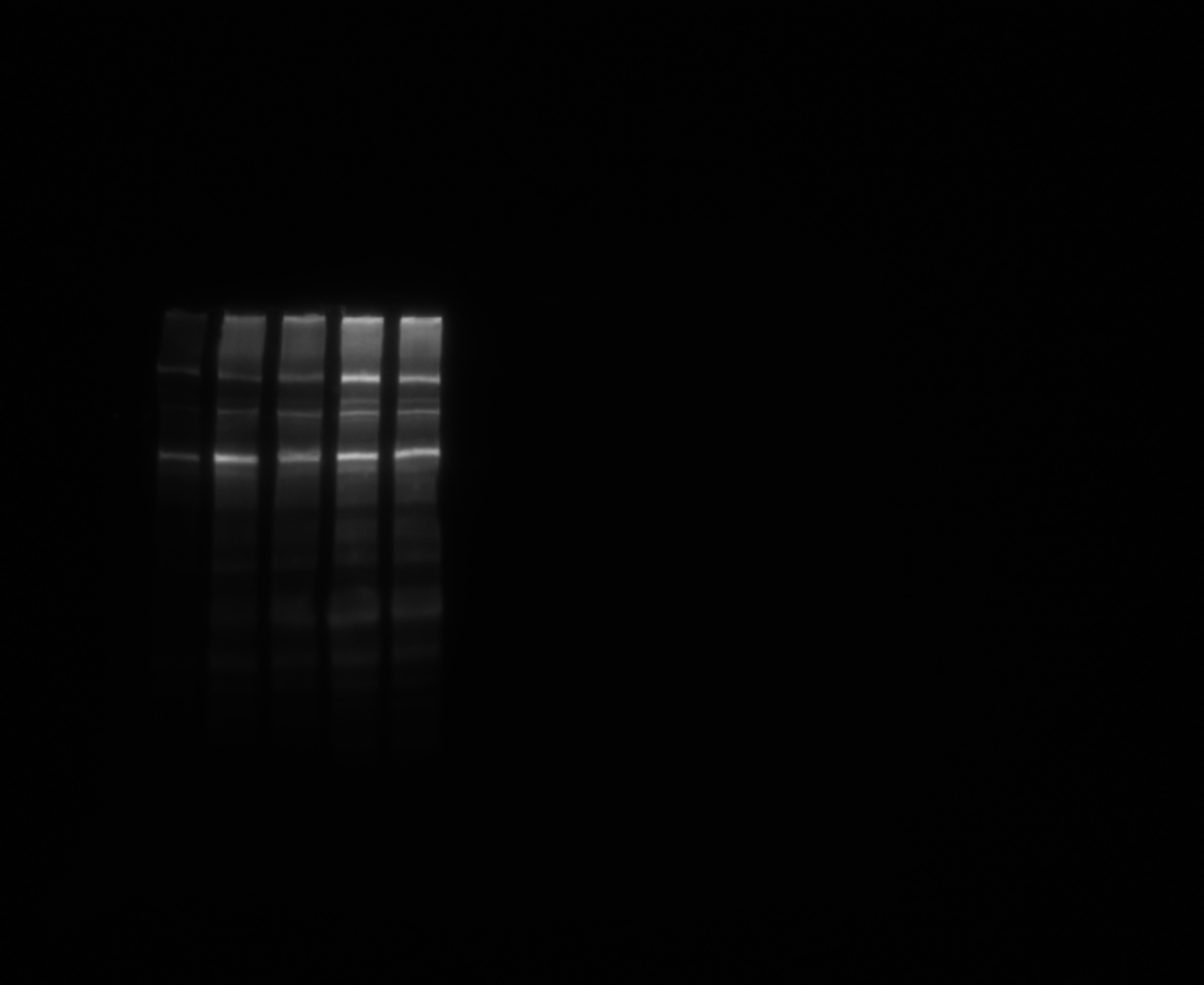

Supplement: Figure 4—source data 3. — Immunoblots for Figure 4G were performed as described in the legend of Figure 4. This folder contains the raw images collected on the Fusion FX Imager (Vilber-Lourmat) for blots probed with anti-dHS and anti-GAPDH antibodies for each of three biological repeats (‘2 reps’ and ‘3rd rep’). [file elife-86931-fig4-data3.zip › Figure 4-source data 3/HDMEC dHS (3rd rep).Tif]

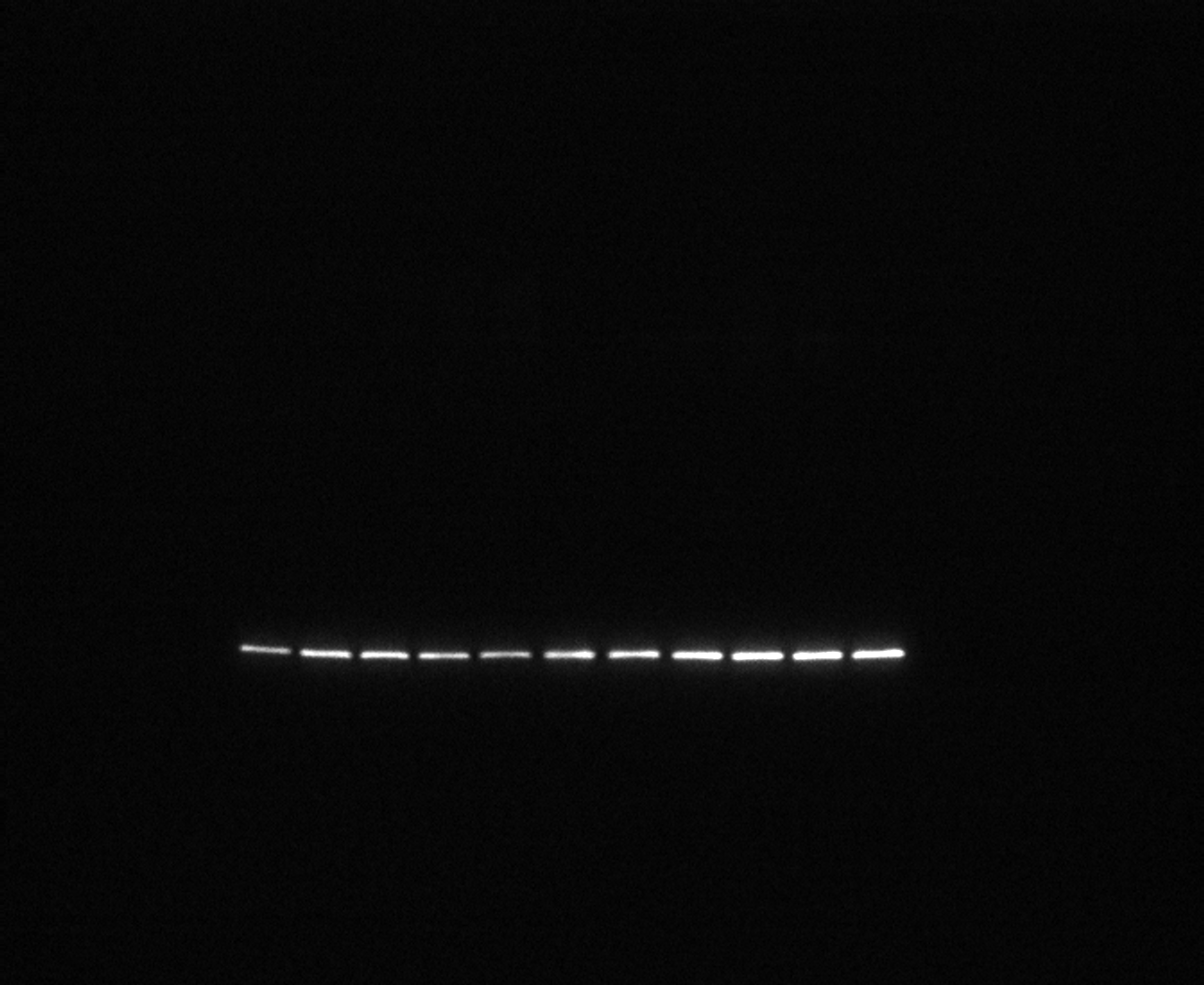

Supplement: Figure 4—source data 3. — Immunoblots for Figure 4G were performed as described in the legend of Figure 4. This folder contains the raw images collected on the Fusion FX Imager (Vilber-Lourmat) for blots probed with anti-dHS and anti-GAPDH antibodies for each of three biological repeats (‘2 reps’ and ‘3rd rep’). [file elife-86931-fig4-data3.zip › Figure 4-source data 3/HDMEC gapdh (2 reps).Tif]

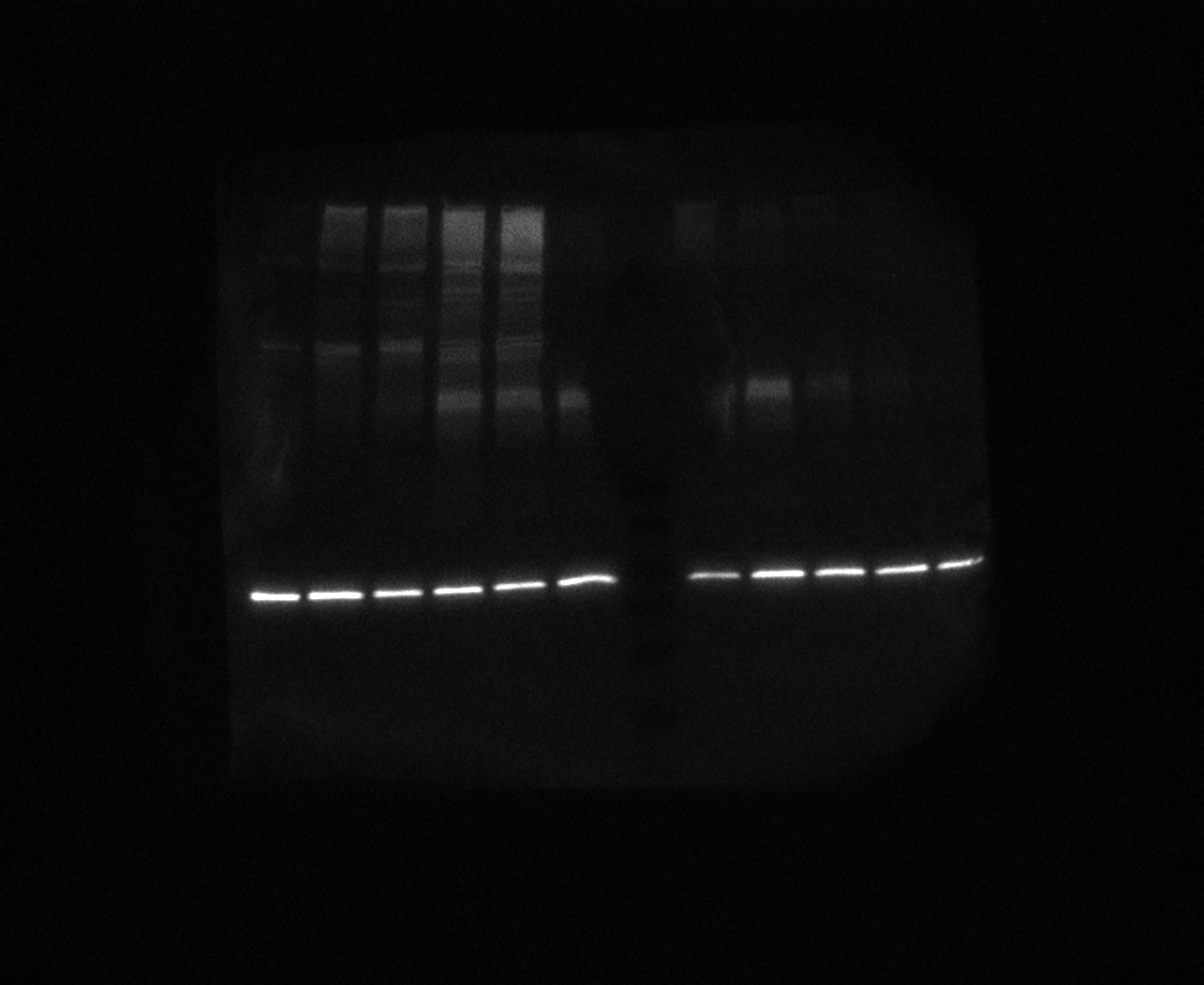

Supplement: Figure 4—source data 3. — Immunoblots for Figure 4G were performed as described in the legend of Figure 4. This folder contains the raw images collected on the Fusion FX Imager (Vilber-Lourmat) for blots probed with anti-dHS and anti-GAPDH antibodies for each of three biological repeats (‘2 reps’ and ‘3rd rep’). [file elife-86931-fig4-data3.zip › Figure 4-source data 3/HDMEC gapdh (3rd rep).Tif]

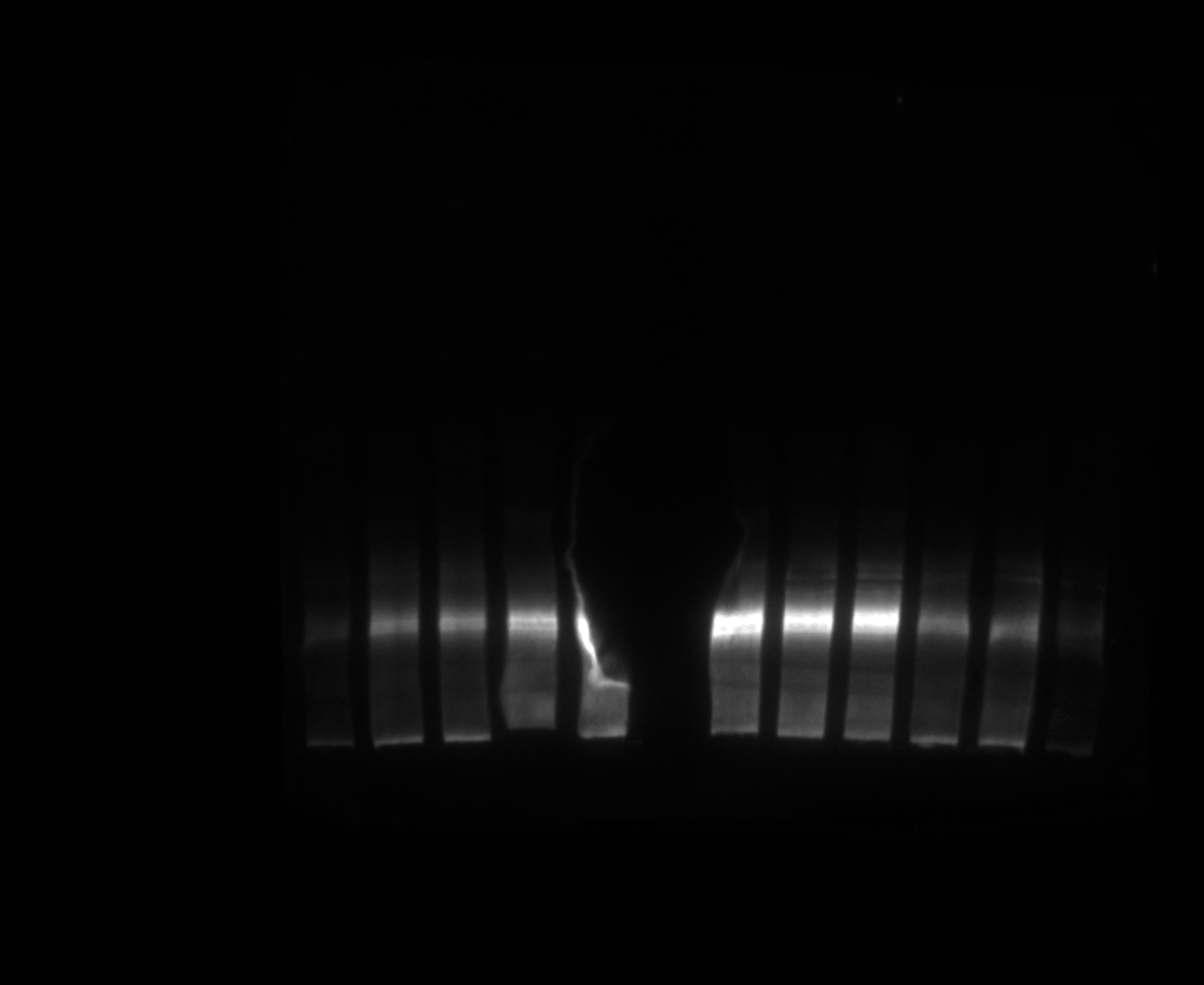

Supplement: Figure 7—source data 2. — Immunoblots for Figure 7—figure supplement 3 were performed as described in the legend of Figure 7—figure supplement 3. This file shows the full-size blots probed with anti-FN1 and anti-GAPDH antibodies for each of three biological repeats (‘rep 1&2’ and ‘rep 3’), alongside molecular weight markers and annotated for treatments. [file elife-86931-fig7-data2.zip › Figure 7-source data 2/1600920 fn2.Tif]

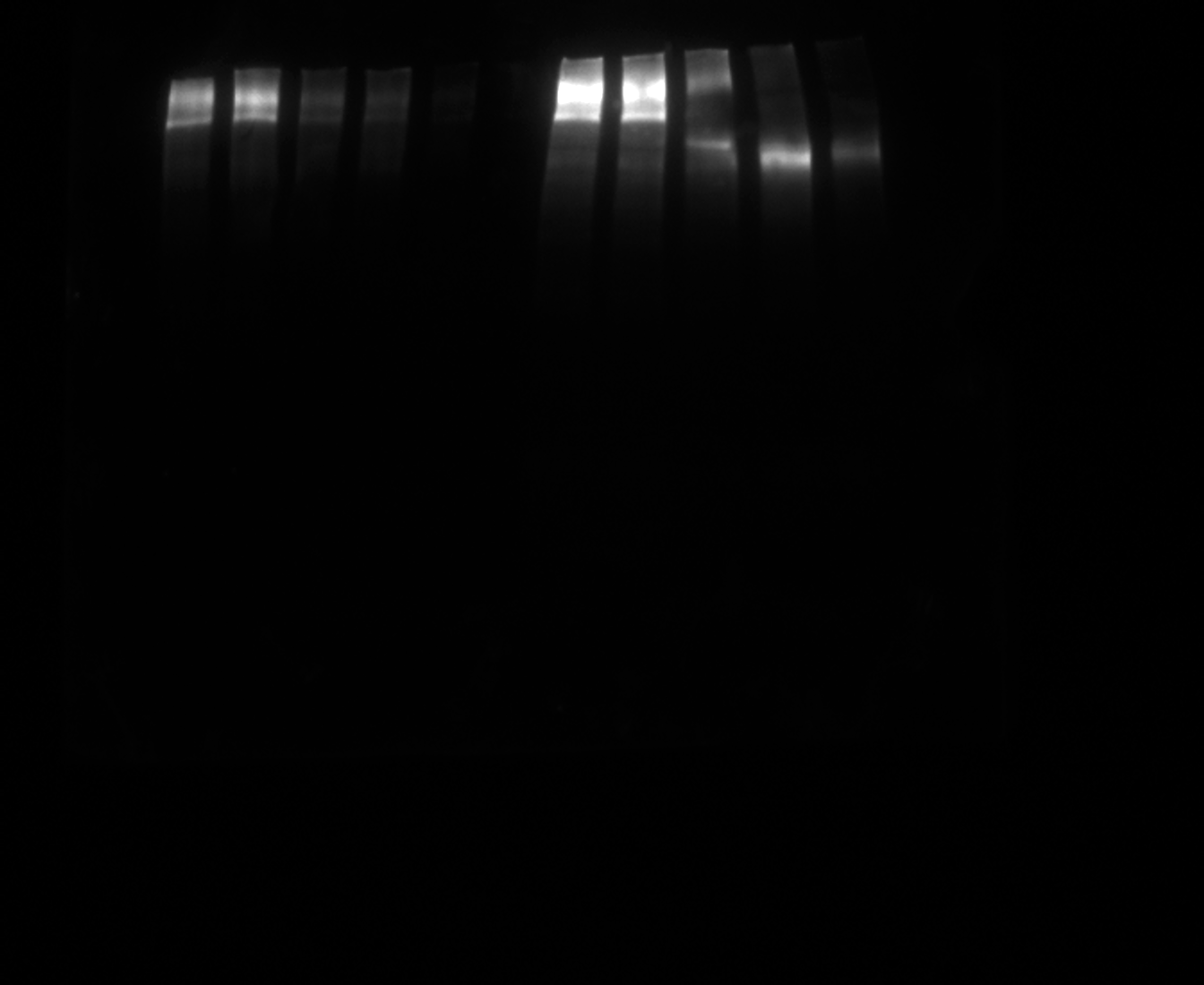

Supplement: Figure 7—source data 2. — Immunoblots for Figure 7—figure supplement 3 were performed as described in the legend of Figure 7—figure supplement 3. This file shows the full-size blots probed with anti-FN1 and anti-GAPDH antibodies for each of three biological repeats (‘rep 1&2’ and ‘rep 3’), alongside molecular weight markers and annotated for treatments. [file elife-86931-fig7-data2.zip › Figure 7-source data 2/160920 fn1.Tif]

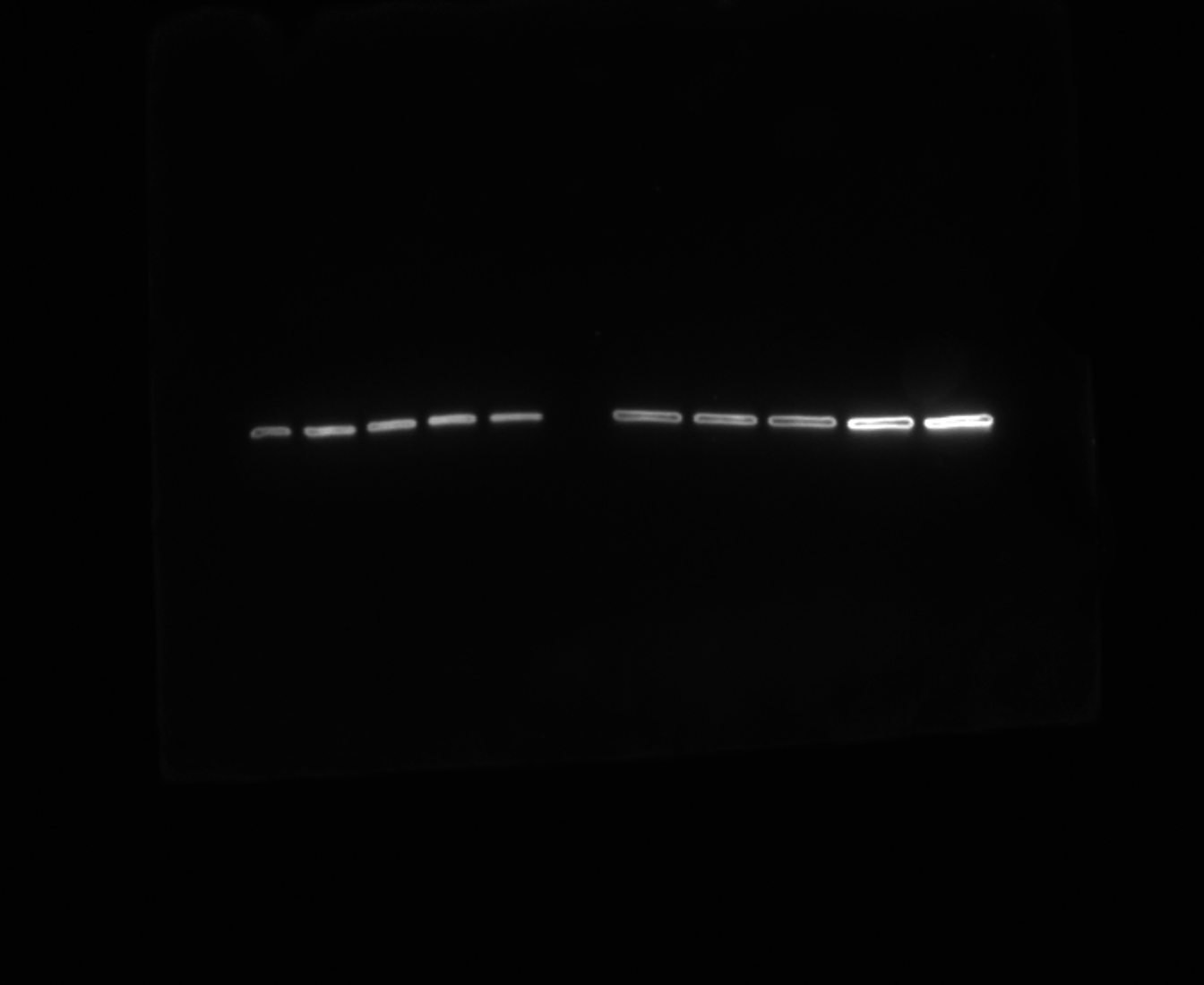

Supplement: Figure 7—source data 2. — Immunoblots for Figure 7—figure supplement 3 were performed as described in the legend of Figure 7—figure supplement 3. This file shows the full-size blots probed with anti-FN1 and anti-GAPDH antibodies for each of three biological repeats (‘rep 1&2’ and ‘rep 3’), alongside molecular weight markers and annotated for treatments. [file elife-86931-fig7-data2.zip › Figure 7-source data 2/170920 hdmec gapdh.Tif]

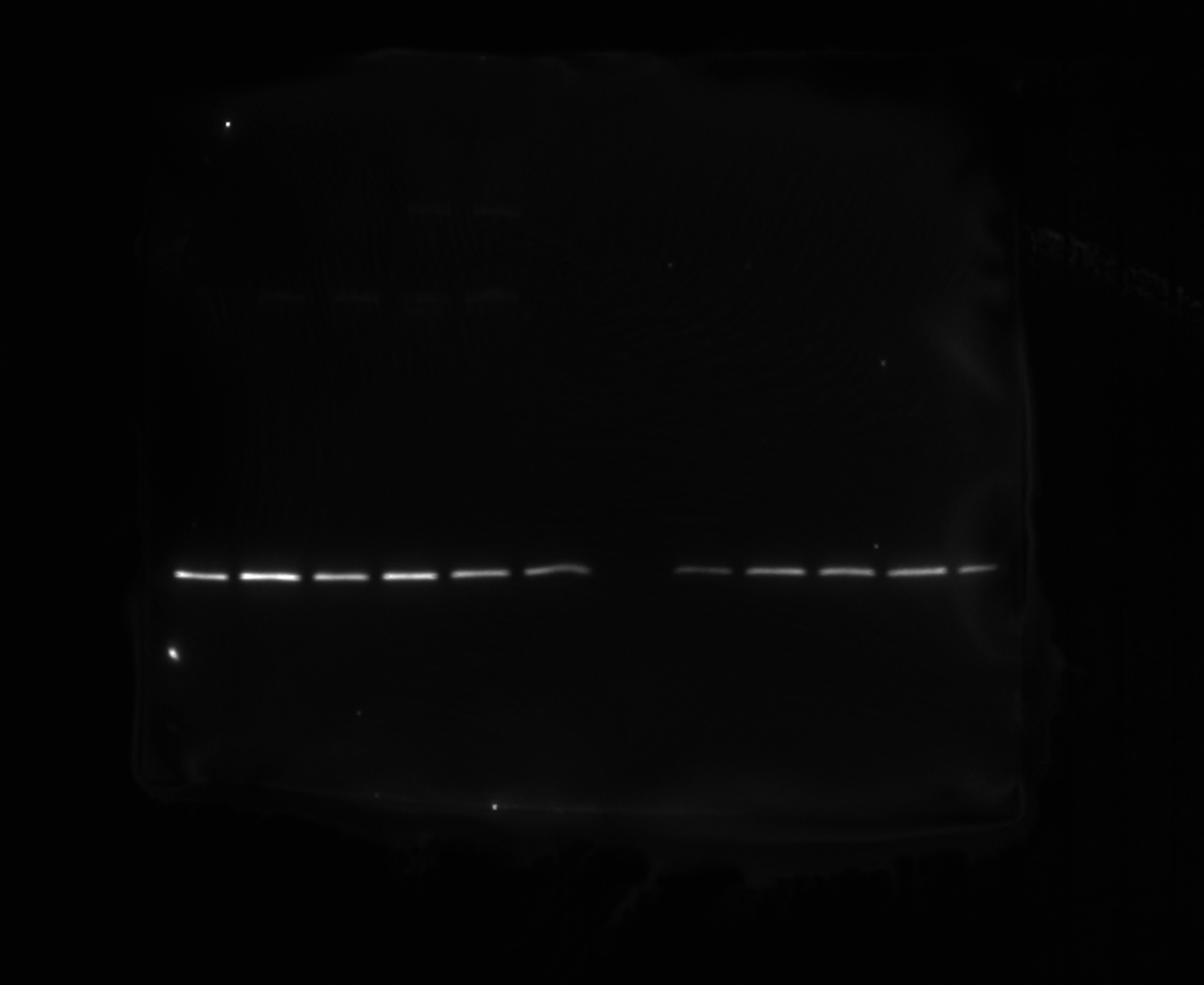

Supplement: Figure 7—source data 2. — Immunoblots for Figure 7—figure supplement 3 were performed as described in the legend of Figure 7—figure supplement 3. This file shows the full-size blots probed with anti-FN1 and anti-GAPDH antibodies for each of three biological repeats (‘rep 1&2’ and ‘rep 3’), alongside molecular weight markers and annotated for treatments. [file elife-86931-fig7-data2.zip › Figure 7-source data 2/170920 hdmec2 gapdh.Tif]

## FIBRONECTIN

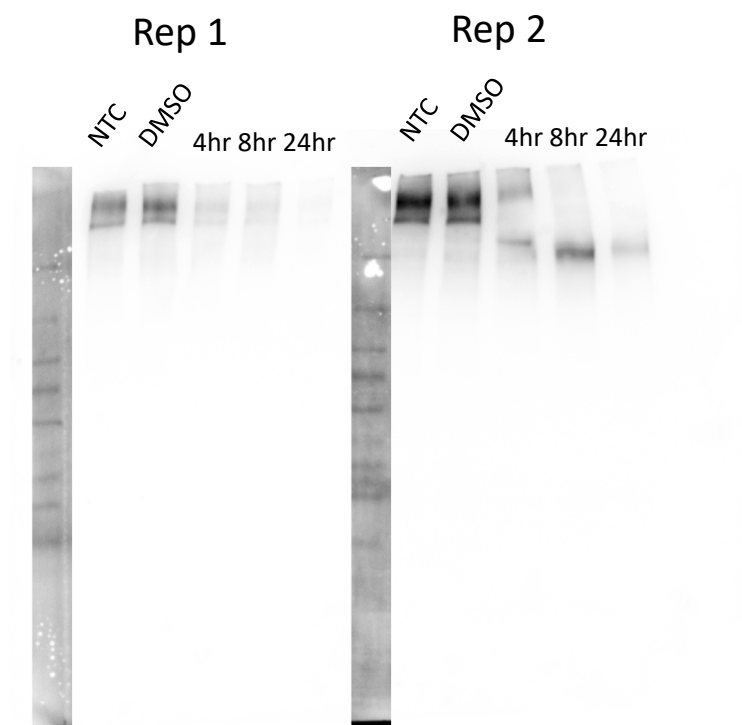

Rep not used

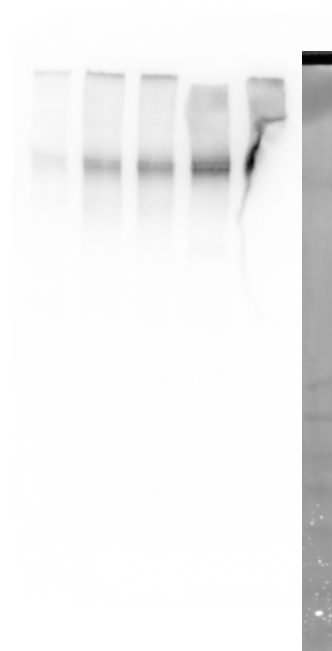

Rep 3

NTC DMSO 4hr 8hr 24hr

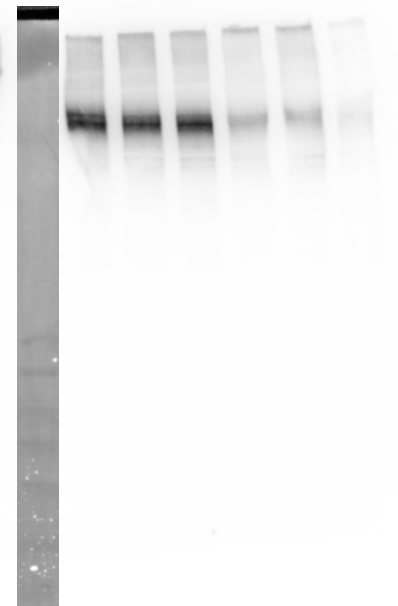

GAPDH

Rep 1

Rep 2

Rep not used

Rep 3

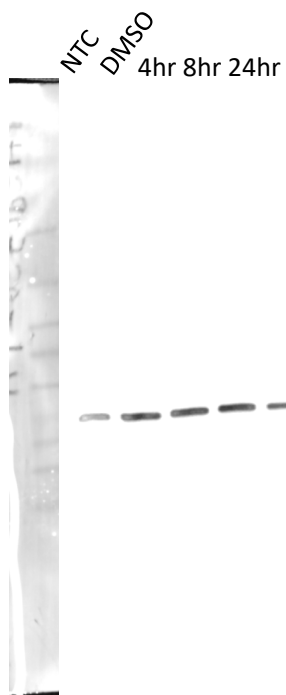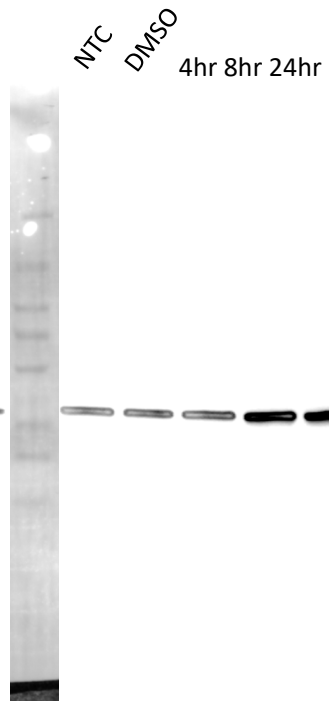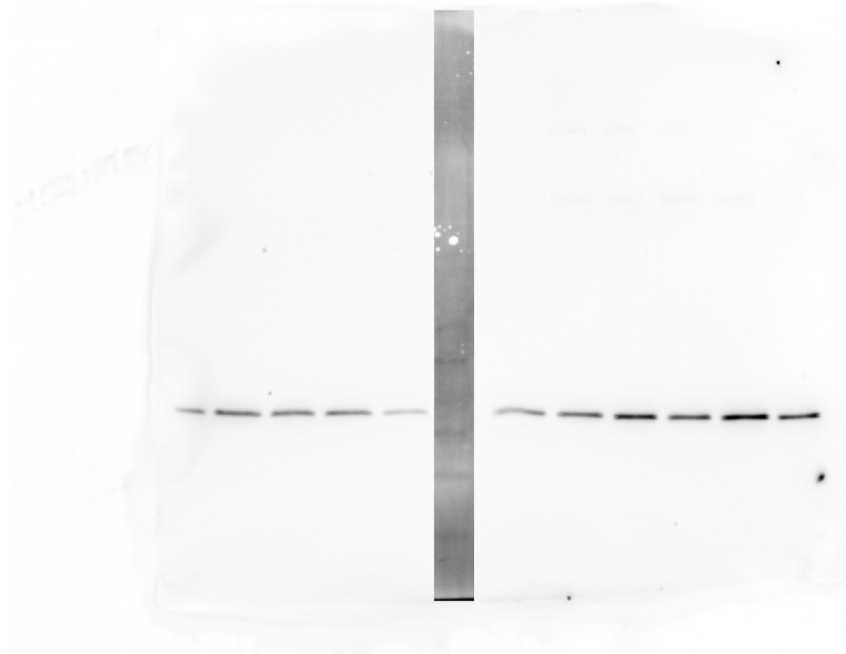

Supplement: Figure 7—source data 2. — Immunoblots for Figure 7—figure supplement 3 were performed as described in the legend of Figure 7—figure supplement 3. This file shows the full-size blots probed with anti-FN1 and anti-GAPDH antibodies for each of three biological repeats (‘rep 1&2’ and ‘rep 3’), alongside molecular weight markers and annotated for treatments. [file elife-86931-fig7-data2.zip › Figure 7-source data 2/Figure 7-figure supplement 3 annotated blots 3 reps.pdf]

ITGA5

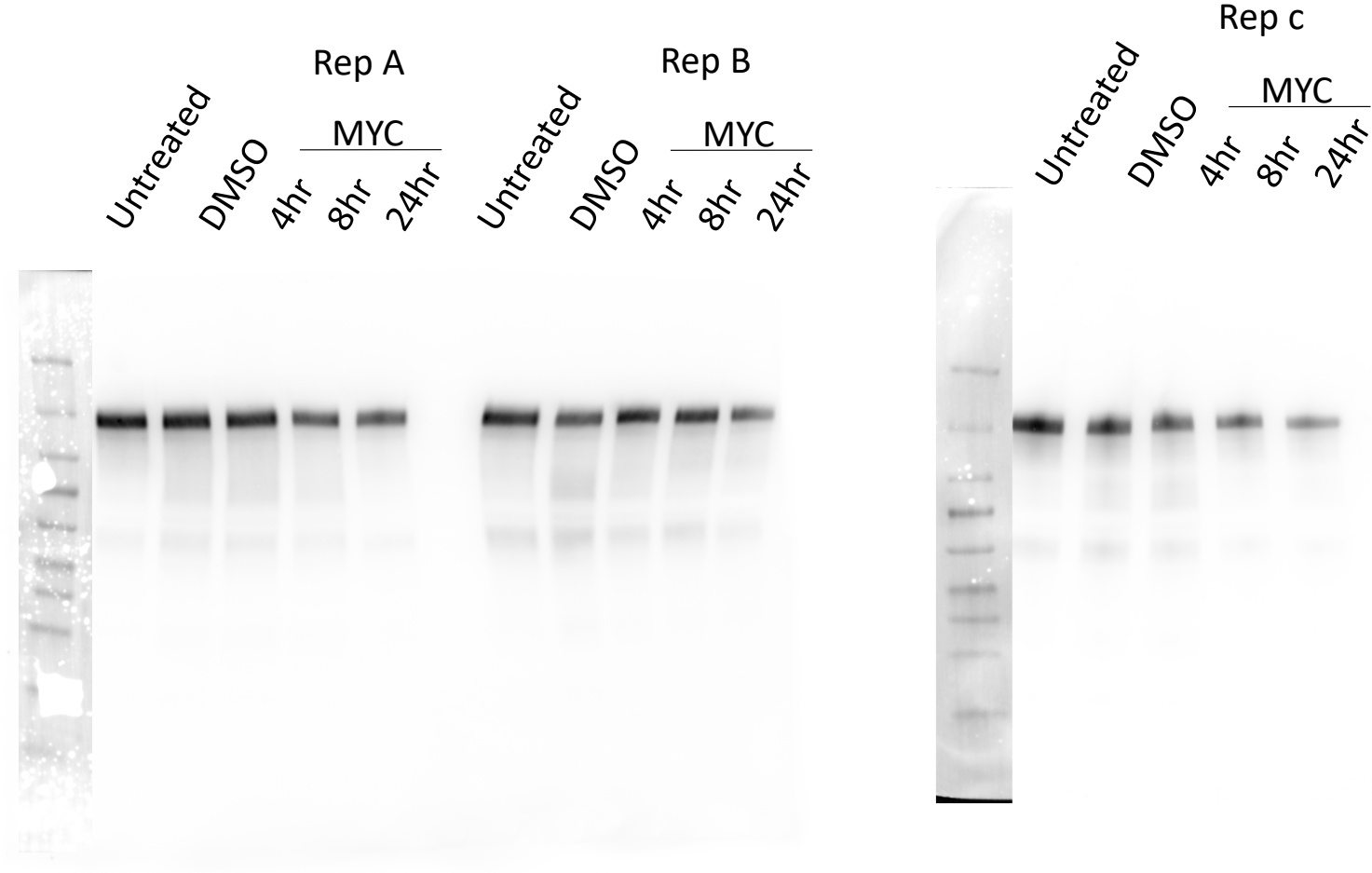

GAPDH

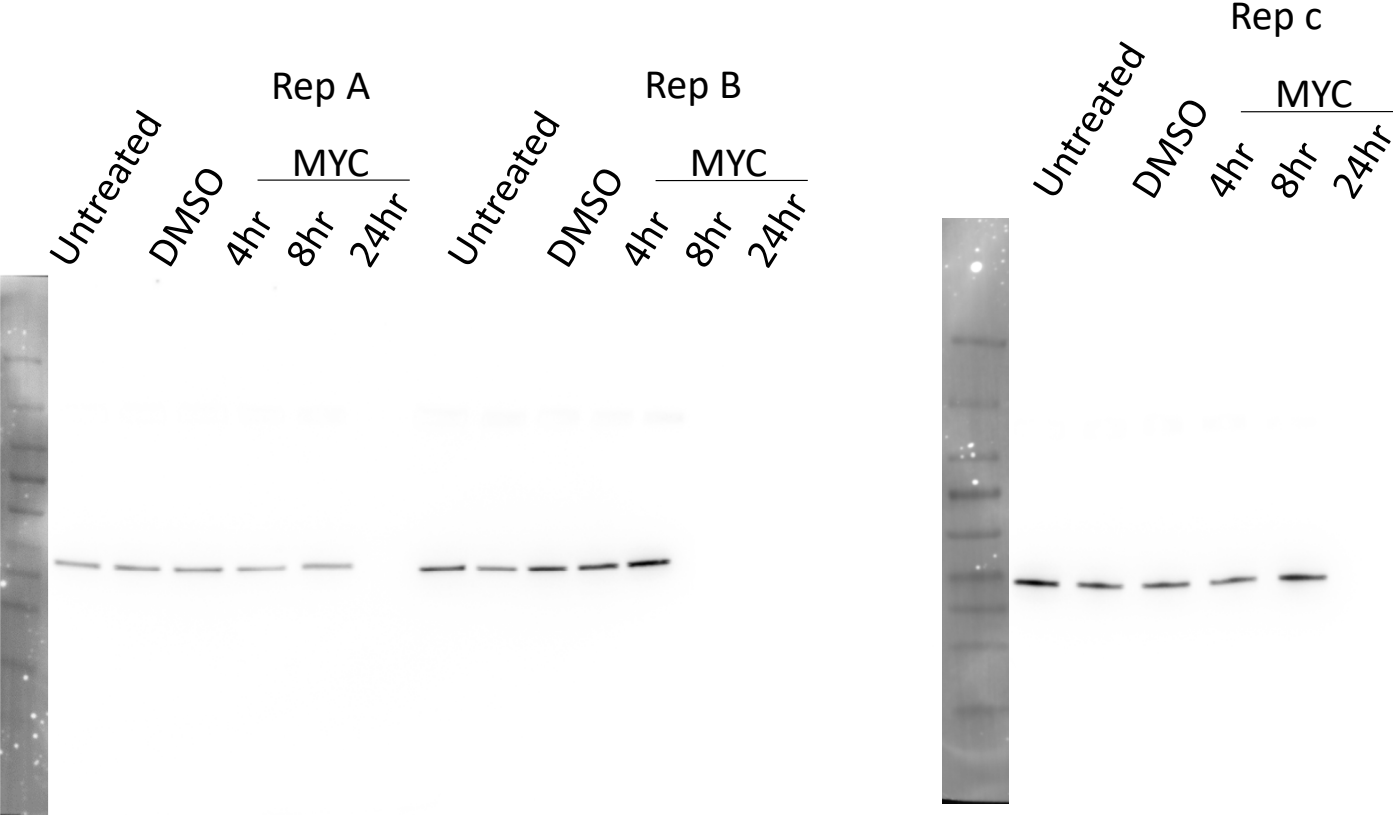

Supplement: Figure 7—source data 4. — Immunoblots for Figure 7—figure supplement 4 were performed as described in the legend of Figure 7—figure supplement 4. This file shows the full-size blots probed with anti-ITGA5 and anti-GAPDH antibodies for each of three biological repeats (‘rep a&b’ and ‘rep c’), alongside molecular weight markers and annotated for treatments. [file elife-86931-fig7-data4.zip › Figure 7-source data 4/Figure 7-source data 3.pdf]

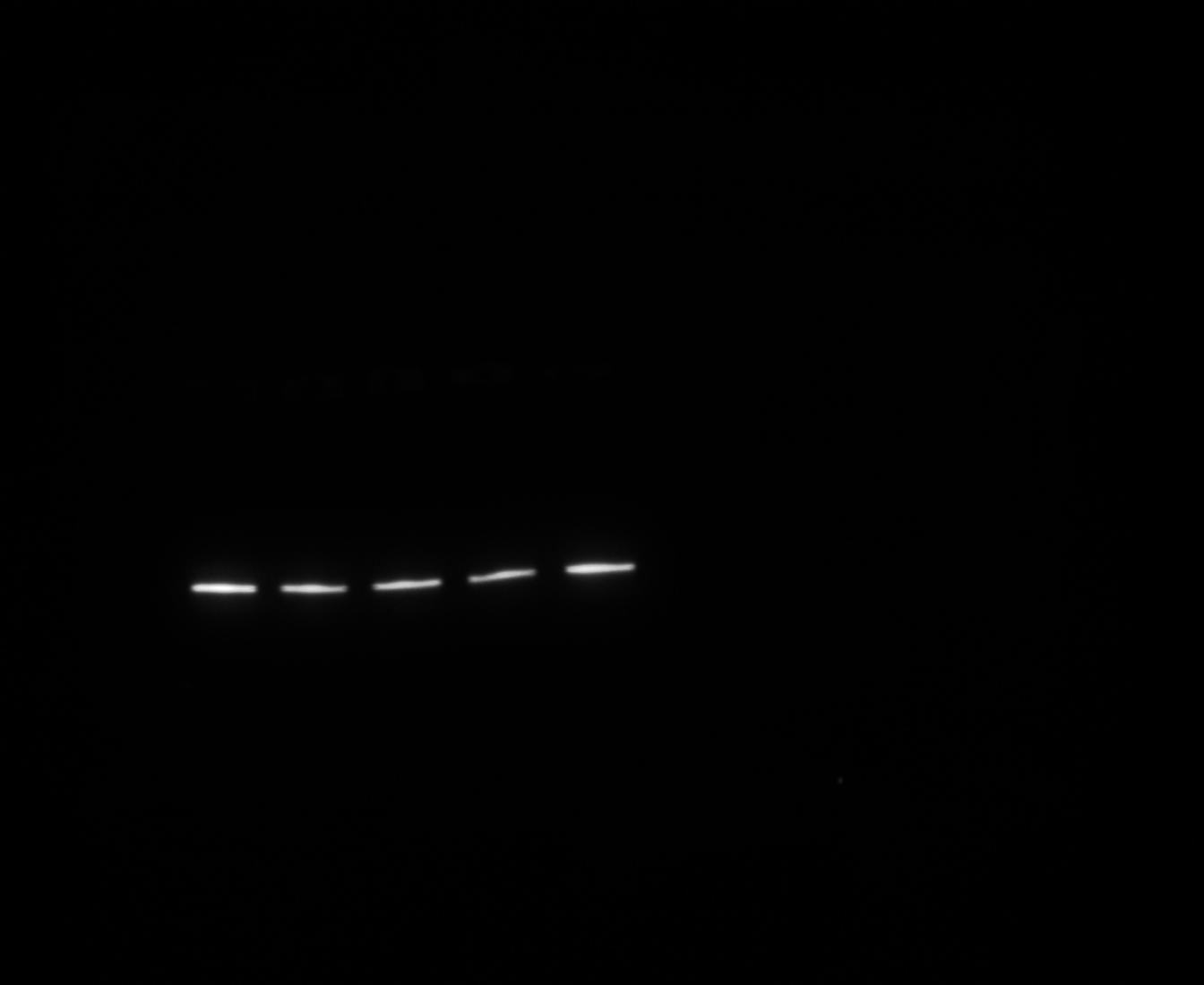

Supplement: Figure 7—source data 5. — Immunoblots for Figure 7—figure supplement 4 were performed as described in the legend of Figure 7—figure supplement 4. This file shows the full-size blots probed with anti-ITGA5 and anti-GAPDH antibodies for each of three biological repeats (‘rep a&b’ and ‘rep c’), alongside molecular weight markers and annotated for treatments. [file elife-86931-fig7-data5.zip › Figure 7-source data 5/hdmec GAPDH rep c.Tif]

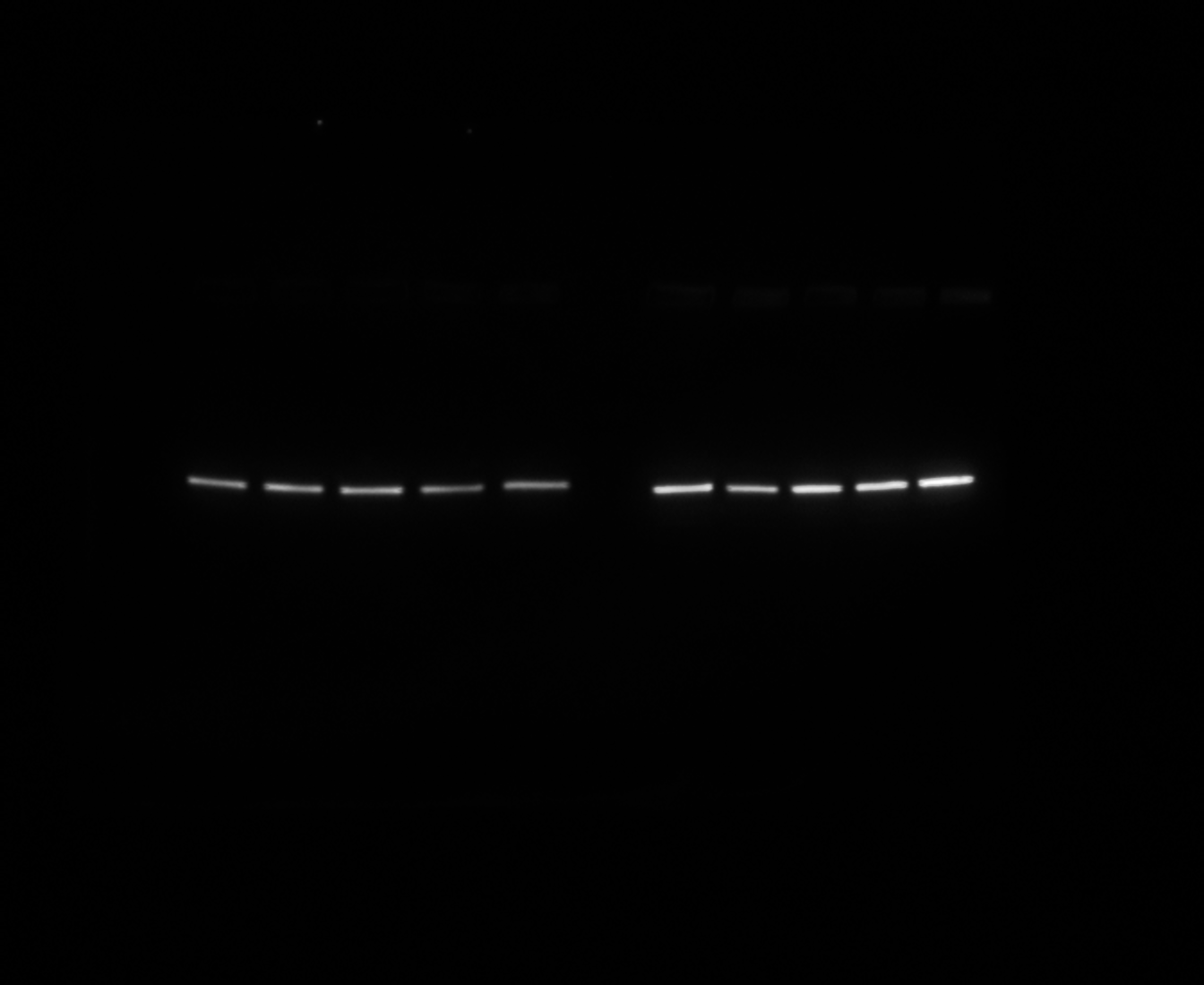

Supplement: Figure 7—source data 5. — Immunoblots for Figure 7—figure supplement 4 were performed as described in the legend of Figure 7—figure supplement 4. This file shows the full-size blots probed with anti-ITGA5 and anti-GAPDH antibodies for each of three biological repeats (‘rep a&b’ and ‘rep c’), alongside molecular weight markers and annotated for treatments. [file elife-86931-fig7-data5.zip › Figure 7-source data 5/hdmec GAPDH reps a&b.Tif]

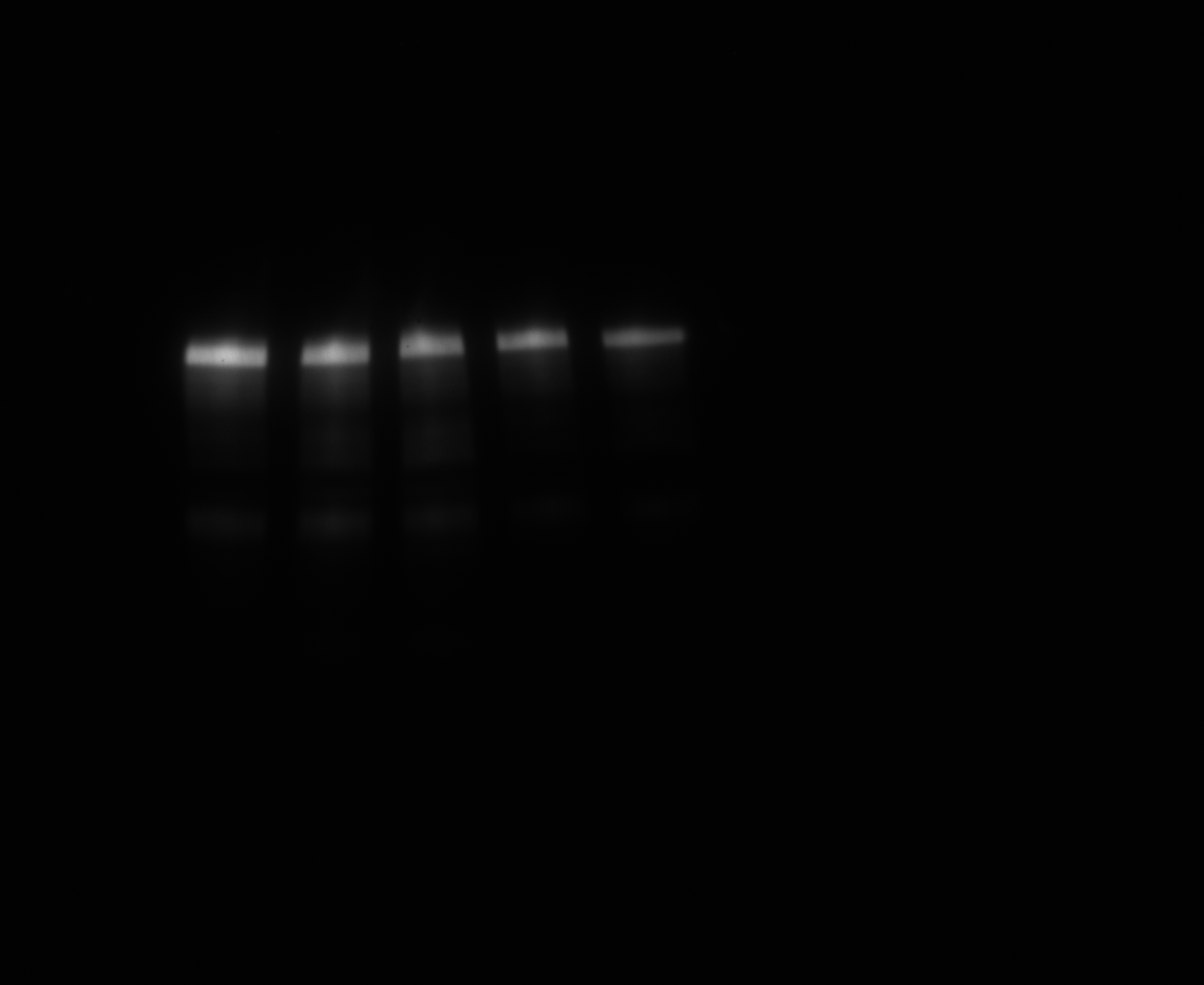

Supplement: Figure 7—source data 5. — Immunoblots for Figure 7—figure supplement 4 were performed as described in the legend of Figure 7—figure supplement 4. This file shows the full-size blots probed with anti-ITGA5 and anti-GAPDH antibodies for each of three biological repeats (‘rep a&b’ and ‘rep c’), alongside molecular weight markers and annotated for treatments. [file elife-86931-fig7-data5.zip › Figure 7-source data 5/hdmec ITGA5 rep c.Tif]

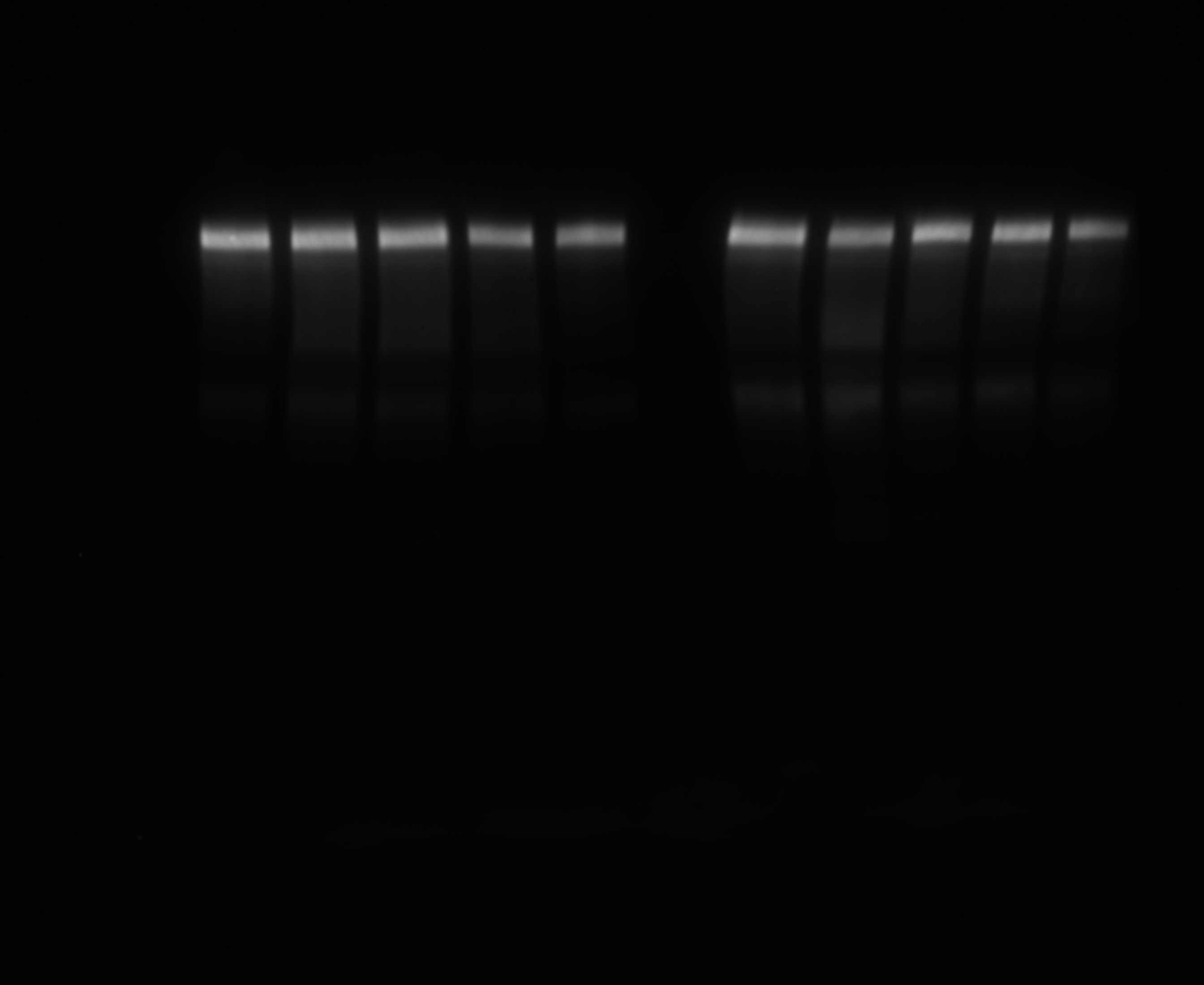

Supplement: Figure 7—source data 5. — Immunoblots for Figure 7—figure supplement 4 were performed as described in the legend of Figure 7—figure supplement 4. This file shows the full-size blots probed with anti-ITGA5 and anti-GAPDH antibodies for each of three biological repeats (‘rep a&b’ and ‘rep c’), alongside molecular weight markers and annotated for treatments. [file elife-86931-fig7-data5.zip › Figure 7-source data 5/hdmec ITGA5 reps a&b.Tif]
